# Synergistic Antiemetic Effects of Nerolidol on Domperidone, Hyoscine, and Ondansetron: *In Vivo* and *in Silico* Investigations on Receptor Binding Affinity

**DOI:** 10.1002/open.202400345

**Published:** 2024-11-12

**Authors:** Sharmita Ghosh Situ, Md. Shimul Bhuia, Raihan Chowdhury, Sakib Al Hasan, Siddique Akber Ansari, Irfan Aamer Ansari, Arman Ali, Muhammad Torequl Islam

**Affiliations:** ^1^ Department of Pharmacy Bangabandhu Sheikh Mujibur Rahman Science and Technology University Gopalganj 8100 Bangladesh; ^2^ Bioinformatics and Drug Innovation Laboratory BioLuster Research Center Ltd., Gopalganj 8100 Dhaka Bangladesh; ^3^ Department of Pharmaceutical Chemistry College of Pharmacy King Saud University Riyadh 11451 Saudi Arabia; ^4^ Department of Drug Science and Technology University of Turin Turin 10124 Italy; ^5^ Pharmacy Discipline Khulna University Khulna Bangladesh

**Keywords:** Emesis, Vomiting, Nerolidol, Molecular docking study.

## Abstract

The present study was designed to measure the potential antiemetic properties of nerolidol (NDL) via *in vivo* and *in silico* studies. To induce emesis copper sulfate pentahydrate (CuSO_4_.5H_2_O) was administered at a dose of 50 mg/kg (orally) to 2‐day‐old chicks. The test sample (NDL) was given at two doses of 50 and 100 mg/kg. b.w. orally. Additionally, aprepitant (16 mg/kg), domperidone (6 mg/kg), hyoscine (21 mg/kg), ondansetron (5 mg/kg), and diphenhydramine (10 mg/kg) were given also orally as positive controls. To observe the modulatory effects of the test sample, combination therapies with reference drugs were also administered to three different groups of animals. Molecular docking and visualization of ligand‐receptor interaction were performed against several emesis‐inducing receptors (5HT_3_, D_2_, D_3_, H_1_, and M_1_‐M_5_) using diverse computational tools. Pharmacokinetics and drug‐likeness of the selected ligands were also calculated. Findings demonstrated that NDL significantly (*p* <0.05) dose‐dependently lessens the mean number of retches and delays the emetic onset in the chicks. The combined drug therapy with ondansetron exposed better antiemetic activity. In addition, *in silico* analysis, NDL has greater binding affinity (−7.3 kcal/mol) against M_2_ and M_3_ receptors. In conclusion, NDL exerted mild antiemetic activity with synergistic properties through muscarinic receptors.

## Graphical abstract

Nerolidol shows prominent antiemetic activity and mitigates CuSO_4_.H_2_O‐mediated retching in chicks efficiently through its peripheral action. Additionally, the *in silico* studies have also shown possible antiemetic effects with greater binding affinity against muscarinic (specially, M_2_ and M_3_) and dopaminergic receptors.

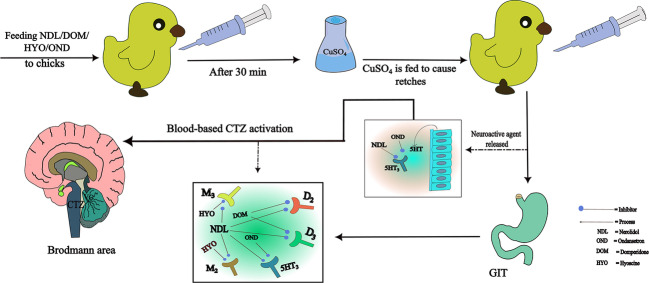

## 1 Introduction

Emesis (also known as vomiting) refers to the forceful ejection of gastric contents, a prominent physiological reflex that can be stimulated by various central and peripheral stimuli.[^1^, ^2^] It characterizes a variety of disorders defined by many weeks of symptoms.^[1]^ It is considered a defense mechanism while aggressive toxins, drugs, or microorganisms come into the body either by the parenteral routes (such as skin, body, inhalation, etc.) or enteral pathways (such as the gastrointestinal tract (GIT)).^[2]^ Separating from this fact, several traumatic events and negative responses to drugs, motion sickness, and other therapeutic situations can also lead to emesis and nausea in the living and natural worlds.^[3]^ Additionally, there are some causes of vomiting, such as gastrointestinal (GI) infections, gastroparesis, migraine, cyclic vomiting syndrome, disturbance of the vestibular, food poisoning, early pregnancy, non‐gastrointestinal effects, etc..[^1^, ^4^, ^5^] However, vomiting is also a familiar side effect of radiation therapy, inhalation, anesthetics, chemotherapy, and opioid analgesics.[^6^, ^7^]

The area postrema (AP), which is also known as the chemoreceptor trigger zone (CTZ), carries receptors that identify emetic agents and transfer that information to the vomiting center (VC). It is responsible for inducing the vomiting response.^[8]^ The brainstem AP lacks the blood brain barrier (BBB), and for disseminating emetic stimuli in the cerebrospinal fluid and blood, it presents as direct central receptor sites.^[9]^ However, orderly administered drugs, which develop sensory signals to the nucleus of the solitary tract, can trigger related receptors present on vegal afferents.[^9^, ^10^] Additionally, peripheral stimuli (for example, microbes, and poisonous drugs) come into the lumen of the GIT and cause the release of confined emetic neurotransmitters (dopamine, serotonin, etc.), which act on connected receptors on vegal afferents, and circulating blood stimulates the CTZ.[^10^, ^11^] Besides CTZ and sensory vegal afferents from the splanchnic nerves (sensation carriers), direct neural inputs are caused by visceral organ disease. Brainstem vestibular nuclei are also responsible for inducing emesis, causing motion sickness.^[12]^ To the dorsal motor nucleus of the vegus, the solitary tract's nucleus has output routes. It promotes the project to the upper GIT to give the vomiting response [9]. On the other hand, the solitary tract's nucleus has projections to the middle and forebrain for the sensitivity of nausea.^[13]^ Some neurotransmitters, such as dopaminergic (D_2_), serotoninergic (5HT_4_, 5HT_3_, and 5HT_1A_), histaminergic (H_1_), neurokinin type 1 (NK_1_), muscarinic acetylcholine (mACh), adrenergic (α_2_), etc., are located in the CTZ and peripheral neural systems (PNS) and are connected to the vomiting response.^[14]^ Additionally, the Gamma Amino Butyric Acid (GABA) receptor is also related to the emetic effects.^[15]^

In the current situation, several antiemetic medications are used in the treatment and management of symptoms associated with vomiting and nausea. These medications are classified based on their mechanisms of action, such as antagonists of dopamine (D_2_ and D_3_), histamine 1 (H_1_), serotonin (5HT_3_), muscarinic (M), corticosteroid, NK_1_ receptors. Among them, NK_1_ and 5HT_3_ antagonists, for example, aprepitant (APR), ondansetron (OND), etc., are more efficient in the treatment of vomiting.^[16]^ However, this current treatment causes some adverse effects, such as dizziness, mild headaches, hypersensitivity, anaphylaxis, dystonia, akathisia, or even life‐hazard complications.^[17]^ Hence, natural products are an alternative form of treatment for diseases. It has contributed to the improvement of current drugs by lowering the adverse effects because of pharmacodynamics and pharmacokinetics (PKs) properties.^[18]^ That's why an ample range of natural compounds, such as cannabinoids, flavonoids, lignans, glucosides, phenylpropanoids, saponins, polysaccharides, triterpenes, sesquiterpenes, etc., are utilized for the development of antiemetic drugs.^[19]^

The nerolidol (NDL), also known as *3,7,11‐trimethyl‐1,6,10‐dodecatrien‐3‐ol*, is a sesquiterpene naturally found in the essential oils of various plants, such as *Momordica charantia* L., *Piper claussenianum (Miq)* C. DC., *Ginkgo biloba* L., *Baccharis dracunculifolia* DC., *Zornia brasiliensis Vogel, Zanthoxylum hyemale* A.St. Hil, and others. This unsaturated acyclic alcohol has two isomers: *cis* and *trans*, and carries a chiral carbon having S and R enantiomers.^[20]^ According to various studies, it shows several pharmacological effects, including, anticancer,^[21]^ anti‐inflammatory,^[22]^ antioxidant,^[23]^ anxiolytic,^[24]^ antinociceptive,^[22]^ antiparasitic,^[25]^ antimalaria,^[26]^ antifungal,^[27]^ antiulcerogenic,^[28]^ anti‐leishmania,^[29]^ repellent,^[30]^ and so on. In a recent study, NDL probably showed an antiemetic effect by blocking the 5‐hydroxytryptamine (5HT) and others signaling pathways and resulting in disruption of emetogenic signaling.^[31]^ Therefore, NDL is a possible natural compound for designing and developing as an antiemetic drug.

There are several *in vivo* and *in vitro* studies available for calculating and evaluating the antiemetic effects of various compounds, and the chick emesis model is one of them.^[32]^ In this model, we used a copper sulfate (CuSO_4_) inducer in young chicks (*Gallus gallus domesticus*), administered orally. The standard is administered for 30 minutes using CuSO_4_ (orally). The antiemetic activity of this experiment is measured by comparing the number of retches and latency with control groups.^[33]^ Additionally, in the field of drug discovery, computer‐aided drug design (CADD) methods have become more important.^[34]^ It allows the prediction of binding affinity and PK properties, which is significant for recognizing and developing probable drug candidates.[^35^, ^36^] Thus, the main objectives of our study are to examine the antiemetic effect of NDL in chicks (in vivo). Moreover, we used computational analysis to investigate molecular interactions that may be responsible for the pragmatic effect as well as the PK properties of NDL.

## 2 Results

### 2.1 *In Vivo* Investigation

**Figure 1:**
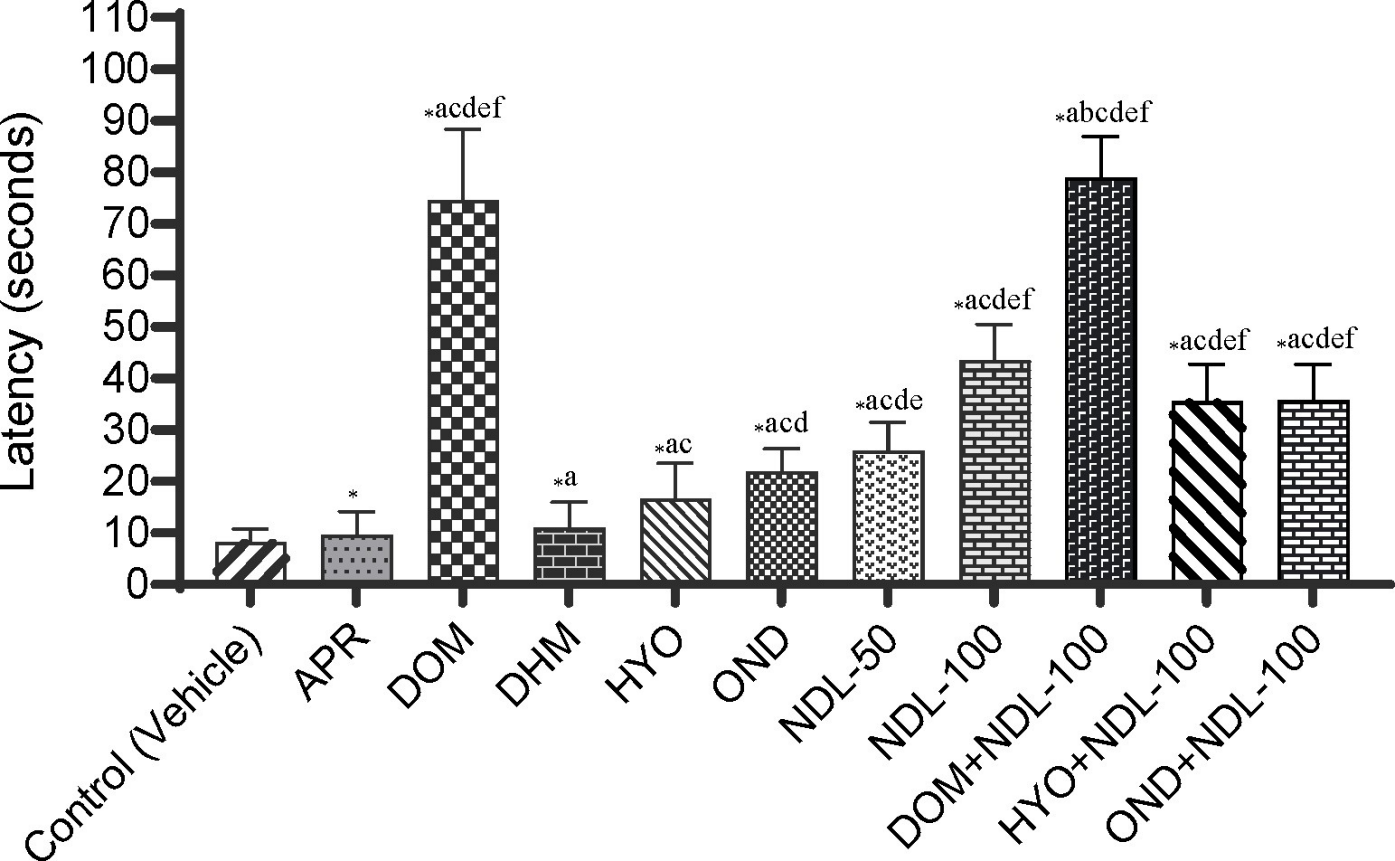
The latency period was identified in test samples, controls, and combinations groups. [Values are the mean ± standard error of the mean (S.E.M.) (n =5)]. p <0.05, ^*^compared to the control (vehicle), ^a^compared to the APR; ^b^compared to the DOM; ^c^compared to the DHM; ^d^compared to the HYO; ^e^compared to the OND; ^f^compared to the NDL‐50. APR: Aprepitant (Dose: 16 mg/kg); DOM: Domperidone (Dose: 6 mg/kg); DHM: Diphenhydramine (Dose: 10 mg/kg); HYO: Hyoscine (21 mg/kg); OND: Ondansetron (Dose: 5 mg/kg) ; NDL‐50: Nerolidol (Dose: 50 mg/kg); NDL‐50: Nerolidol (Dose: 50 mg/kg); DOM+NDL‐100: Domperidone+Nerolidol; HYO+NDL‐100: Hyoscine+Nerolidol; OND+NDL‐100: Ondansetron+Nerolidol.

**Figure 2:**
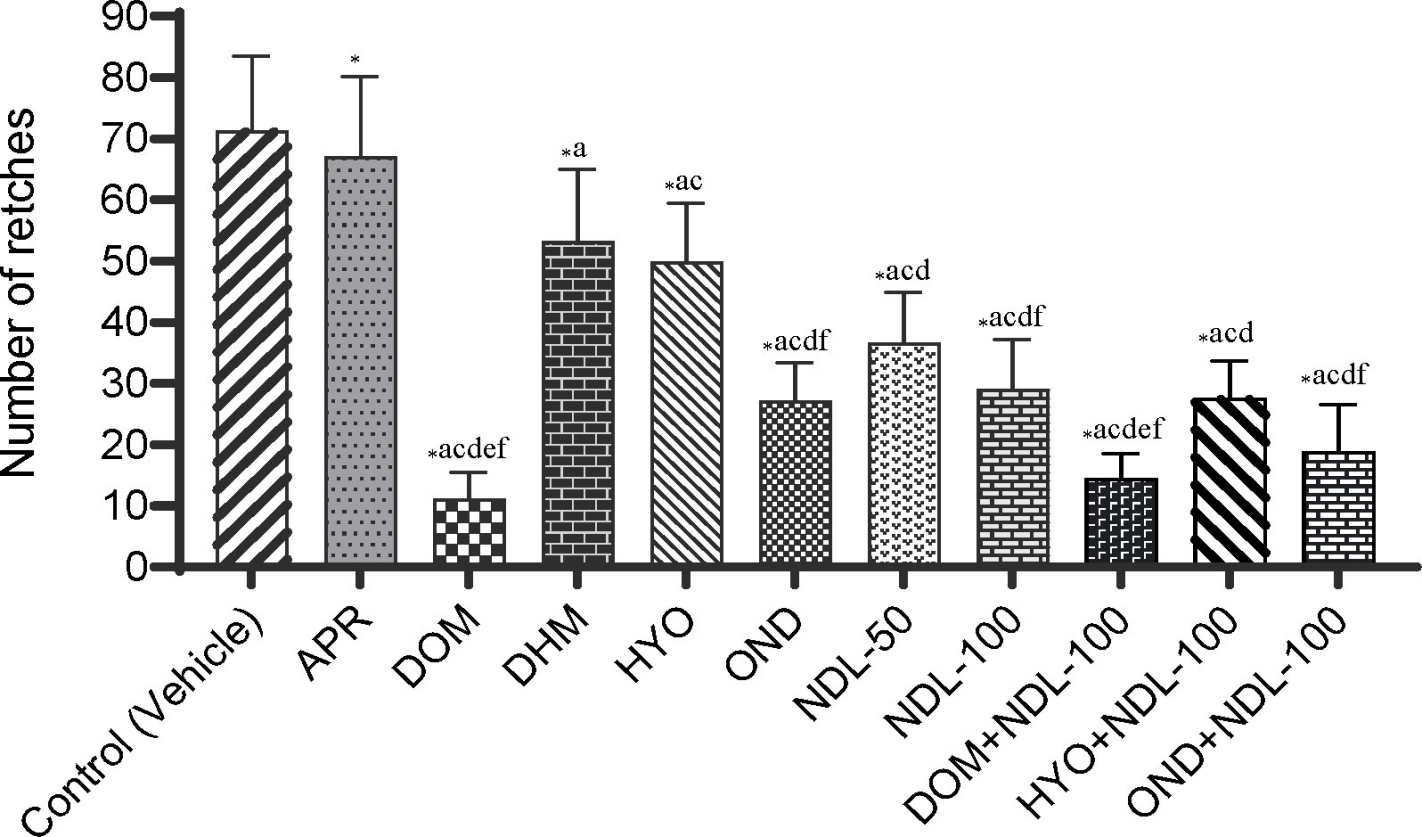
The number of retches was found in the test sample, controls, and combination groups. [Values are mean ± standard error of the mean (SEM) (n =5). p<0.05, ^*^compared to the control (vehicle), ^a^compared to the APR; ^b^compared to the DOM; ^c^compared to the DHM; ^d^compared to the HYO; ^e^compared to the OND; ^f^compared to the NDL‐50. APR: Aprepitant (Dose: 16 mg/kg); DOM: Domperidone (Dose: 6 mg/kg); DHM: Diphenhydramine (Dose: 10 mg/kg); HYO: Hyoscine (21 mg/kg); OND: Ondansetron (Dose: 5 mg/kg); NDL‐50: Nerolidol (Dose: 50 mg/kg); NDL‐100: Nerolidol (Dose: 100 mg/kg); DOM+NDL‐100: Domperidone+ Nerolidol; HYO+NDL‐100: Hyoscine+ Nerolidol; OND+NDL‐100: Ondansetron + Nerolidol; Control (vehicle): Distilled water (Dose:10 mL/kg)].

In our test, in the control group (vehicle), animals showed their first latency at 8.33±0.99 sec, while animals in the reference groups exhibited a prominent latency compared to the control group. The highest latency (74.50±5.66 sec) was observed in the domperidone (DOM) group in animals among the reference drugs mentioned in this experiment. The onset of retching values for reference groups is 9.67±1.84, 11.00±2.05, 16.67±2.81, and 21.83±1.85 sec for aprepitant (APR), diphenhydramine (DHM), hyoscine (HYO), and ondansetron (OND), respectively (Figure 1). In addition, in comparison to the control group, animals in the experimental groups (NDL) experienced a significant dose‐dependent elevation in latency. The NDL‐100 group showed the highest latency (43.50±2.86 sec) among the entire test group, except for the DOM and DOM+NDL‐100 groups, while the other experimental group (NDL‐50) was exposed to 26.00±2.24 sec. The combination therapies demonstrated that NDL remarkably increased latency when the animals were co‐treated with the reference drugs in comparison to the reference drugs alone. The latency of the DOM+NDL‐100, OND+NDL‐100, and HYO+NDL‐100 groups is 79.00±3.21, 35.50±2.96, and 35.67±2.89 sec, respectively. Our obtained latency for all tested groups is described in Figure 1. In the vehicle group (71.33±4.96 sec) of this experiment, the highest number of retches was exhibited. With reference to drugs such as APR, DOM, DHM, HYO, and OND, a noteworthy reduction in the number of retches was observed in animals in comparison to the control group. The DOM group exposed the lowest retches (11.17±1.78 sec) among the selected reference drugs, still crosswise among all the treatment groups. The number of retching values for APR, DHM, HYO, and OND are 67.17±5.31, 53.33±4.75, 50.00±3.89, and 27.17±2.55 sec, respectively. However, there was a significant dose‐dependent reduction in the number of retches in the test sample, and animals in the NDL‐50 and NDL‐100 groups showed 36.67±3.35 and 29.17±3.25 sec, respectively. In the combination therapies, the DOM+NDL‐100 (14.50±1.65 sec) group exposed the lowest number of retches. The total number of retches and latency for all experimental groups is given in Figures 1 and 2.

According to Table 1, the NDL‐50 and NDL‐100 groups have an increased percentage of latency in comparison to the vehicle group of animals, which was 67.96 and 80.85 %, respectively. Outcomes indicated that the latency period increased with the amplification of doses in the experimental groups. Though the combination treatments in animals exhibited a significant elevation in the latency percentage, among the several combination treatments, DOM+NDL‐100 showed the highest latency percentage of 89.45 %. On the other hand, a percentage decrease in retches demonstrated a dose‐dependent percentage reduction in retching. The maximum percentage reduction in retching was experimented with in the DOM group (84.34 %). However, the combination therapy of drugs with NDL exhibited a decrease in retching, which was 79.67 %. Our outcomes revealed a decrease in retching percentage for other combination groups, which were 61.20 and 73.60 % for the HYO+NDL‐100 and OND+NDL‐100, respectively. The percentage augment in latency and the decline in retching for each experimental group are displayed in Table 1.

**Table 1:** Percentage increase in latency and decrease of retching in emetic animals of the experiment and control groups.

Treatment Groups	Increase in Latency (%)	Decrease in retches (%)
Control (Vehicle)	–	–
APR	13.86	5.83
DOM	88.82	84.34
DHM	24.27	25.23
HYO	50.03	30.00
OND	61.84	61.90
NDL‐50	67.96	48.59
NDL‐100	80.85	59.10
DOM+NDL‐100	89.45	79.67
HYO+NDL‐100	76.54	61.20
OND+NDL‐100	76.65	73.60

Control (vehicle): Distilled water (dose: 10 mL/kg); APR: Aprepitant (dose: 16 mg/kg); DOM: Domperidone (dose: 6 mg/kg); DHM: Diphenhydramine (dose: 10 mg/kg); HYO: Hyoscine (dose: 21 mg/kg); OND: Ondansetron (dose: 5 mg/kg); NDL‐50: Nerolidol (dose: 50 mg/kg); NDL‐100: Nerolidol (dose: 100 mg/kg); DOM+NDL‐100: Domperidone+Nerolidol; HYO+NDL‐100: Hyoscine+Nerolidol; OND+NDL‐100: Ondansetron + Nerolidol.

### 2.2 *In silico* Investigation

#### 2.2.1 Homology Modelling of the Human 5HT_3_ Protein

**Figure 3:**
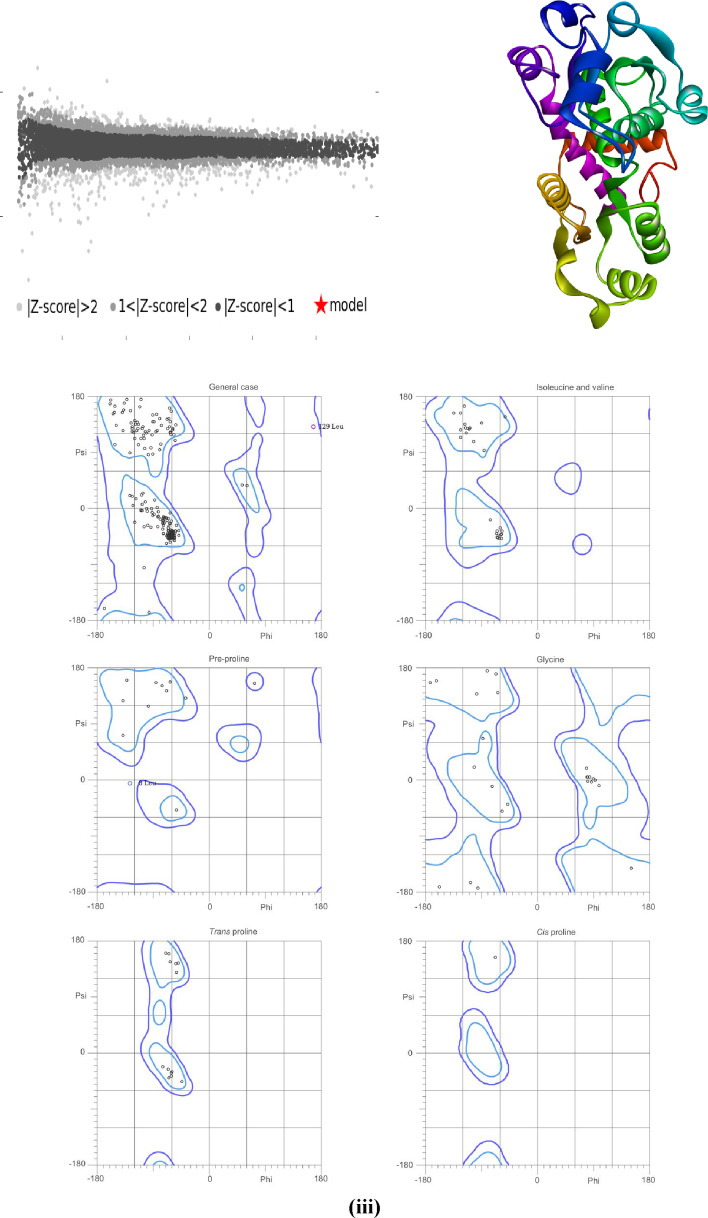
i) Ramachandran plot of the homology modeled 5HT3 protein, ii) 3D structure of human 5HT3 receptor homology model build by SWISS MODEL, iii) Comparison between the modelled protein structure and a non‐redundant set of PDB structures.

From the outcomes of homology modeling, it exhibits the desired sequence and template sequence of 8BL8 (PDB ID), a 3D structure of the human 5HT_3_ receptor,^[37]^ have an almost similar sequence with a template of an X‐ray method, 2 Å (99.34 % identity with the template sequence). With a QMEAN of 0.93 and a GMQE score of 0.95, the homology model of 5HT_3_ was constructed. It suggests high quality and consistency. For the verification of consistency and accuracy of the residues Psi and Phi angles, the Ramachandran plot was developed. The Ramachandran plot exhibited two outliers (8 Leu and 129 Leu) and 96.9 % Ramachandran favorable in Figure 3. 
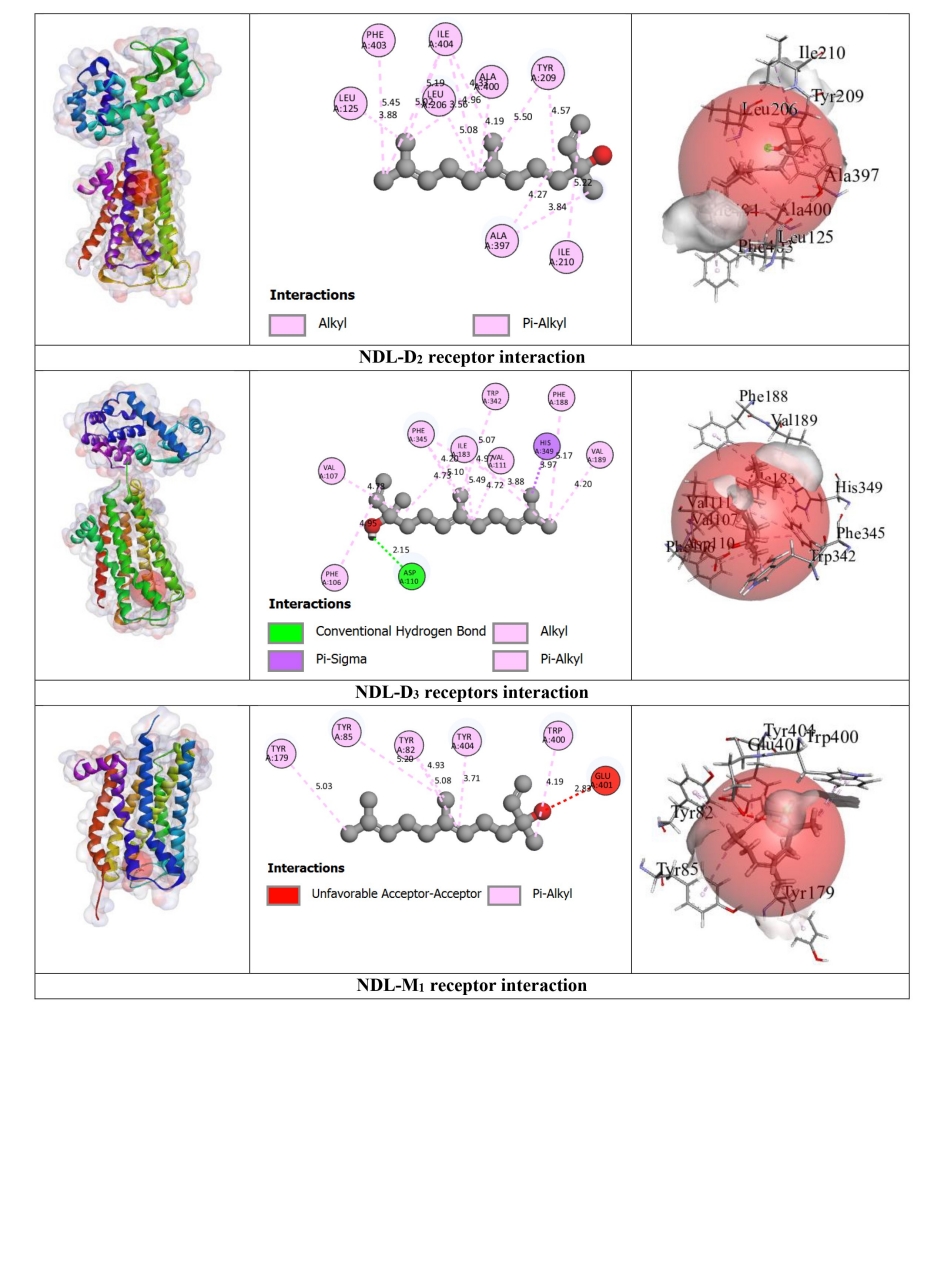
 
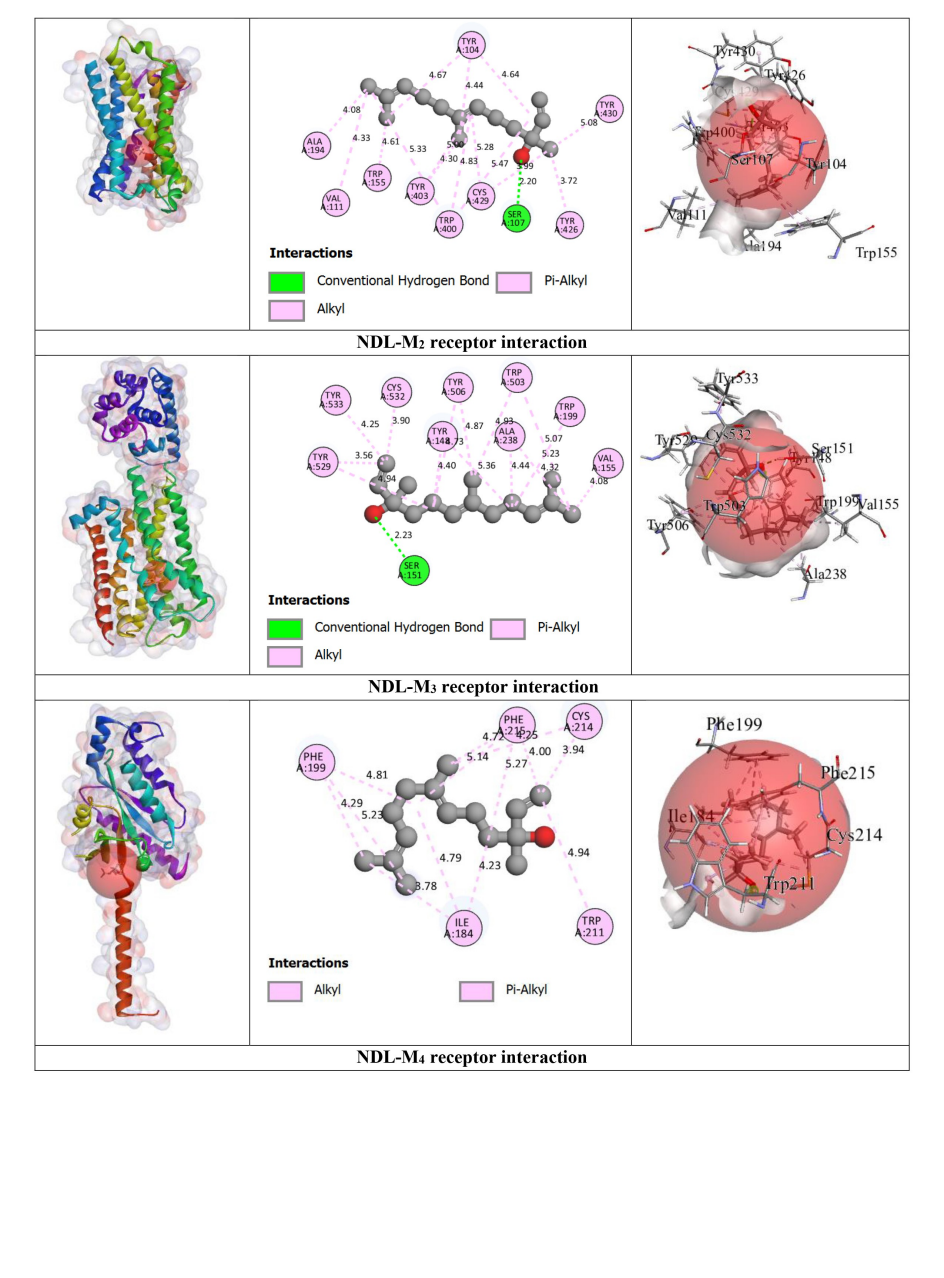
 
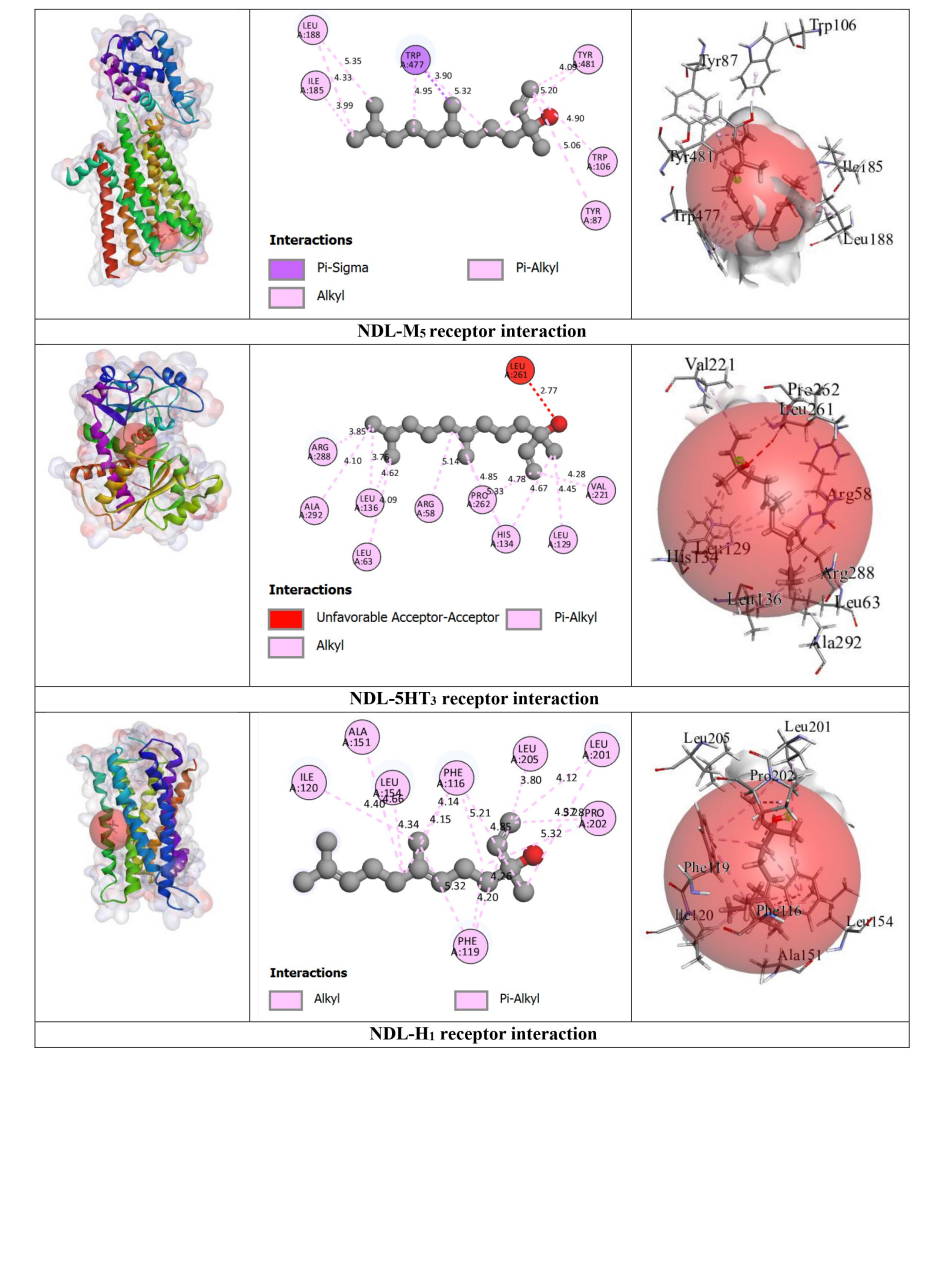
 
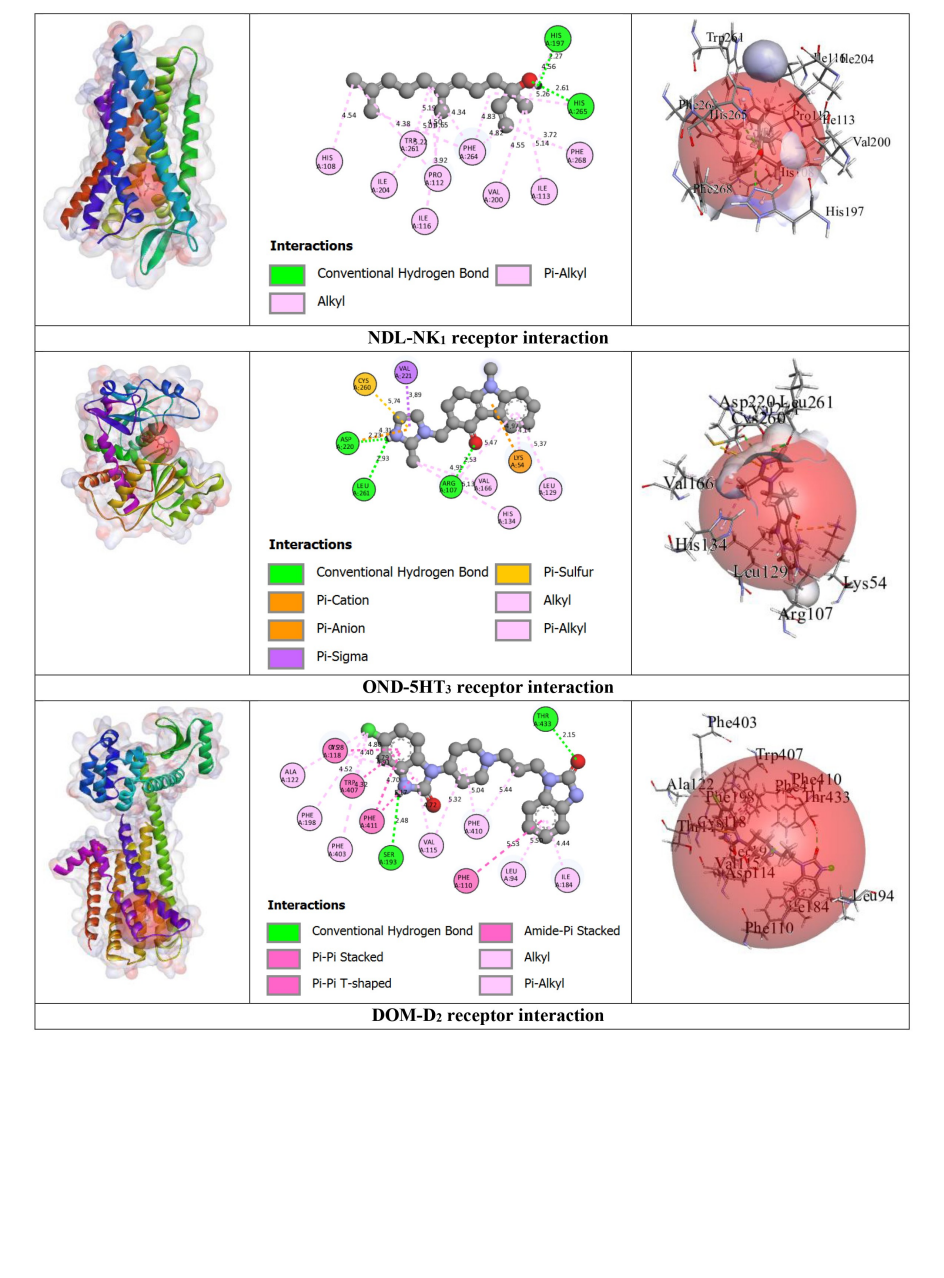


#### 2.2.2 Molecular Docking

For the prediction of probable binding affinity energy between ligand and protein, a molecular docking analysis was utilized. From our *in silico* result, the test ligand (NDL) exhibits the highest docking score with both the M_2_ and M_3_ receptors; the value was −7.3 kcal/mol for both. These selected emesis‐mediated receptors (M_2_ and M_3_) show docking scores of −7.5 and −8.9 kcal/mol with the reference ligand, HYO. The NDL ligand also exhibited a higher docking score with the M_5_ subunit (−6.2 kcal/mol) apart from M_1_ and M_4_ (Table 2). NDL also illustrated binding affinity with D_2_ and D_3_ receptors; their highest docking scores are −6.5 kcal/mol for both. On the other hand, the chosen reference ligand, DOM, shows the highest binding affinity with D_2_ and D_3_ receptors, and the outcomes are −10.6 and −8.5 kcal/mol, respectively. Additionally, the 5HT_3_ exposes a −7 kcal/mol docking score with the selected drug OND. NDL towards the H_1_ emesis mediating subunit shows lower binding scores (−5.8 kcal/mol), while H_1_ with the chosen reference drug, DHM, shows a better binding score (−6.3 kcal/mol). However, APR binds with the NK_1_ receptor by exhibiting an outstanding binding score of −12.6 kcal/mol, while NDL shows a lower binding interaction (−6.5 kcal/mol). The docking scores for test ligands and drugs used aligned with the particular receptors are shown in Table 2.

**Table 2:** Molecular docking scores (kcal/mol) of selected ligands against different proteins liable for inducing emesis.

**Ligands**	**Receptors**										
		**D_2_**	**D_3_**	**5HT_3_**	**M_1_**	**M_2_**	**M_3_**	**M_4_**	**M_5_**	**NK_1_**	**H_1_**
	**PDB ID**	6LUQ	3PBL	–	6WJC	5ZK8	4 U15	7 V6 A	6OL9	6HLO	3RZE
NDL		−6.5	−6.5	−5.4	−5.2	−7.3	−7.3	−5	−6.2	−6.5	−5.8
DOM		−10.6	−8.5	–	–	–	–	–	–	–	–
OND		–	–	−7	‐	–	–	–	–	–	–
HYO		–	–	–	−8.6	−7.5	−8.9	−5.8	−7.5	–	–
DHM		–	–	–	–	–	–	–	–	–	−6.3
APR		–	–	–	–	–	–	–	–	−12.6	–

Molecular docking score is presented in kcal/mol; APR: Aprepitant, DOM: Domperidone, DHM: Diphenhydramine, HYO: Hyoscine, OND: Ondansetron, NDL: Nerolidol, D_1_‐D_2_: Dopaminergic, 5HT_3_: Serotoninergic, H_1_: Histaminergic, NK_1_: Neurokinin type 1, and M_1_‐M_5_: Muscarinic receptors

#### 2.2.3 Prediction of Non‐Bond Interactions Between Protein‐Ligand Complexes

**Figure 4:**
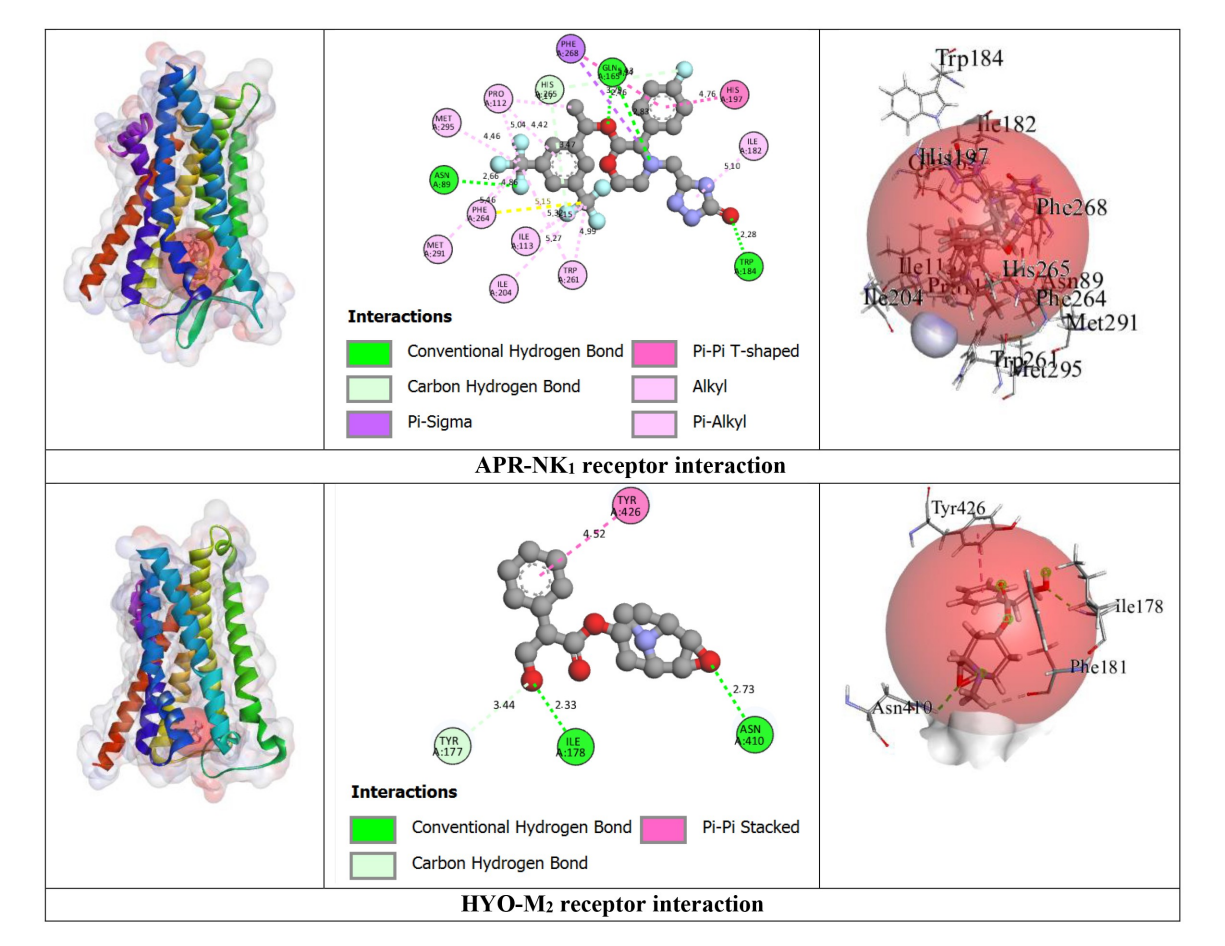
3D view of receptor binding site and 2D view of interacted amino acid (AA) residues between chosen ligands and several emesis‐inducing receptors.

Our outcomes from the *in silico* study reveal that ligands interact with receptors via initiating several types of bonds, which include hydrogen bond (HB), hydrophobic bond (HPB), and other types of bonds together with alkyl, pi‐alkyl, sigma, pi‐sulphur, pi‐carbon, pi‐pi T‐shaped, etc. For the M_2_ and M_3_ receptors, NDL exhibited a greater docking score value of −7.3 kcal/mol for both in comparison to other receptor interactions with NDL. NDL combines with the M_2_ receptor via establishing two HBs; they are SER107 and CYS429 with various HPB with amino acid (AA) residues of VAL111, ALA194, TYR104, TRP155, TRP400, TYR403, TYR426, and TYR430, respectively. However, NDL binds with the M_3_ receptor by forming one HB (SER151) and a variety of other bonds with AA residues of CYS532, VAL155, ALA238, TYR148, TRP199, TRP503, TYR506, TYR529, and TYR533. On the other hand, M_1_, M_4_, and M_5_ receptors do not show any HB but form HPB with NDL ligands. DOM shows better antagonizing action against D_2_ and D_3_ receptors via forming 6HB and 3HB, specifically SER193, THR433, and ASP114 for D_2_ and THR369, SER366, and GLY93 for D_3_, and the highest docking score of −10.6 and −8.5 kcal/mol. Both of them show several other bonds, particularly SER193, ASP114, PHE110, PHE411, TRP407, ALA122, VAL115, CYS118, LEU94, and ILE184 for the D_2_ receptor, and SER366, GLY93, VAL107, and VAL86 for the D_3_ receptor, respectively. But in the case of NDL, it forms no HB with D_2_ and one HB with the D_3_ receptor, particularly ASP110, which shows −6.5 kcal/mol for both. In the case of the OND and 5HT_3_ receptors interaction, it displays a −7 kcal/mol docking score with 4HB, specifically ASP220, LEU261, ARG107, and LEU261; and several HPBs. However, there is no HB or variety of HPB between NDL and the 5HT_3_ receptor. On the other hand, the result finds no HB bond in both interactions of NDL and H_1_ receptor and DHM and H_1_ receptor with the binding affinity of −5.8 and −6.3 kcal/mol, respectively. Additionally, the highest binding affinity is found in the interaction between APR and NK_1_ receptor (−12.6 kcal/mol) with 6 HBs, namely, ASN89, GLN165, TRP184, and HIS265 AA residues, and several other bonds (mainly HPB), specifically, ASN89, GLN165, TRP184, HIS265, HIS197, TRP261, and PHE264. However, the interaction between NDL and NK_1_ exposes a binding affinity of −6.5 kcal/mol for forming 3HB, particularly HIS197 and HIS265 and a variety of HPB. Altogether, it is noticeable that NDL showed the highest binding affinity against the M_2_ and M_3_ receptors among all emesis‐mediating receptors. The number of HB, HPB, HB length, AA residues, types of bonds, etc. is given in Table 3 and Figure 4.

**Table 3:** Amino acid residues, number of hydrogen bonds, and hydrogen bond length of non‐bond interactions between the chosen ligands and receptors.

Proteins	Ligands	No. of HB	No. of HPB	HB residues	HB length (Å)	Other bond residue
D_2_	DOM	6	18	SER193, THR433, ASP114.	2.14, 2.56, 2.63, 3.03, 1.64.	SER193, ASP114, PHE110, PHE411, TRP407, ALA122, VAL115, CYS118, LEU94, ILE184.
	NDL	0	14	–	–	LEU206, ILE404, ALA397, ILE210, LEU125, ALA400, TYR209, PHE403.
D_3_	DOM	3	4	THR369, SER366, GLY93	2.77, 3.06, 2.66.	SER366, GLY93, VAL107, VAL86.
	NDL	1	13	ASP110.	2.14.	VAL111, ILE183, VAL107, VAL189, PHE106, PHE188, TRP342, PHE345, HIS349.
5HT_3_	OND	4	10	ASP220, LEU261, ARG107, LEU261.	2.72, 2.92, 2.52, 2.63.	LYS54, ASP220, CYS260, VAL166, VAL221, ARG107, LEU129, HIS134.
	NDL	0	12	–	–	ARG58, HIS134, LEU129, VAL221, PRO262, LEU136, ARG288, ALA292.
M_1_	HYO	2	5	ASP105	2.42, 2.27.	ASP105, VAL113, ALA196.
	NDL	0	7	–	–	TYR82, TYR85, TYR179, TRP400, TYR404.
M_2_	HYO	4	1	ILE178, ASN410, PHE181, TYR426.	2.32, 2.72, 2.71, 2.27.	TYR426.
	NDL	2	15	SER107, CYS429.	2.19, 3.98.	VAL111, ALA194, TYR104, TRP155, TRP400, TYR403, TYR426, TYR430
M_3_	HYO	5	7	TYR148, ASP147, TYR529, THR234.	2.70, 2.98, 2.27, 2.86, 2.96.	TYR148, CYS532, TYR148, TRP503, TYR506, TYR529, ALA238.
	NDL	1	14	SER151.	2.23.	CYS532, VAL155, ALA238, TYR148, TRP199, TRP503, TYR506, TYR529, TYR533.
M_4_	HYO	1	3	–	2.27.	ASP337, TYR320, ALA338, PHE334.
	NDL	0	14	–	–	ILE184, CYS214, PHE199, TRP211, PHE215.
M_5_	HYO	4	2	LEU188, SER189, TYR111.	2.93, 2.52, 2.27, 2.91.	TRP477.
	NDL	0	12	–	–	ILE185, LEU188, TYR87, TRP106, TRP477, TYR481.
NK_1_	APR	6	17	ASN89, GLN165, TRP184, HIS265.	2.66, 2.83, 2.45, 2.27, 3.46, 3.34.	ASN89, GLN165, TRP184, HIS265, HIS197, TRP261, PHE264.
	NDL	3	16	HIS197, HIS265.	2.26, 2.61, 3.03.	PRO112, ILE116, ILE204, ILE113, VAL200, HIS108, HIS197, TRP261, PHE264, HIS265, PHE268.
H_1_	DHM	0	6	–	–	PHE116, PHE119, PRO202, ILE120, ALA151.
	NDL	0	15	–	–	LEU154, ILE120, PRO202, LEU201, LEU205, ALA151, PHE116, PHE119,

APR: Aprepitant, DOM: Domperidone, DHM: Diphenhydramine, HYO: Hyoscine, OND: Ondansetron, NDL: Nerolidol, D_1_‐D_2_: Dopaminergic, 5HT_3_: Serotoninergic, H_1_: Histaminergic, NK_1_: Neurokinin type 1, M_1_‐M_5_: Muscarinic, HB: Hydrogen bond, HPB: Hydrophobic bond

#### 2.2.4 Estimation of *In Silico* Pharmacokinetics and Drug‐Likeness

**Figure 5:**
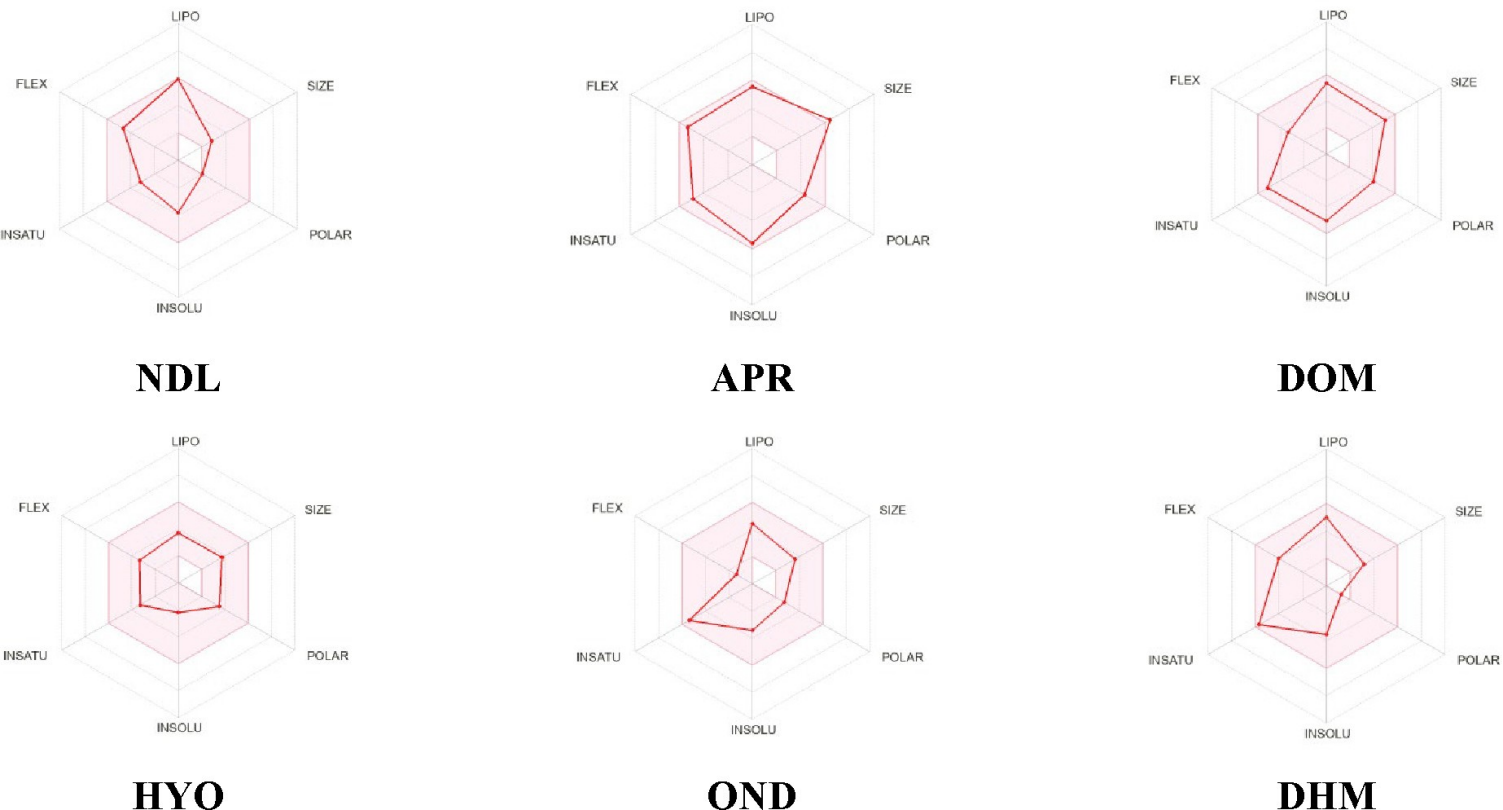
Summary of physiochemical, toxicological, and pharmacokinetics properties of selected compounds. [The colored zone is the suitable physicochemical space for oral bioavailability; SIZE within 150 g/mol<MV<500 g/mol; INSOLU (Insolubility) within −6<logS(ESOL)<0; LIPO (Lipophilicity) within −7<XLOGP3<+5.0; INSATU (In saturation) within 0.25<Fraction Csp3<1; POLAR (Polarity) within 20, Å^2^<TPSA<130 Å^2^; FLEX (Flexibility) within 0<num. rotatable bonds<9; NDL: Nerolidol; APR: Aprepitant, DOM: Domperidone, DHM: Diphenhydramine, HYO: Hyoscine, OND: Ondansetron.]

PKs and drug‐likeness parameters are essential features of a drug candidate and involve improving a chemical substance into a drug. The *in silico* absorption, distribution, metabolism, elimination, and toxicity (ADMET) process plays important roles in drug development and discovery. Molecular weight (MW), molecular formula (MF), hydrogen bond acceptors (HBAs), hydrogen bond donors (HBDs), and molar refractivity (MR) are the main parameters by which drug‐likeness can be measured. Our investigating outcomes demonstrated that except for APR, all drugs have MW below 500 Dalton. Based on Lipinski's rule of five, apart from APR, the values of HBAs for all drugs are within equal or below 10 and HBDs are within equal or below 5. Despite APR and HYO, there is a possibility for them to pass via the BBB. The MR values of NDL, DOM, HYO, OND, DHM, and APR are 74.00, 124.08, 83.48, 87.39, 79.10, and 118.82, respectively. Except for APR, all drugs exhibit higher GI absorption. Additionally, DOM, HYO, and APR show no CYP1 A2 inhibitors, but others show CYP1 A2 inhibitors. All the drugs expose Ghose, Veber, and Lipinski, but only APR shows Ghose. Hence, the bioavailability scores (BS) for all drugs are 0.55. The estimated values of various PK and physicochemical parameters are also given in Table 4 and Figure 5.

**Table 4:** Several parameters of drug‐likeness and pharmacokinetics of nerolidol and selected standards measured by SwissADME.

Properties	Parameters	NDL	DOM	HYO	OND	DHM	APR
Physicochemical and water solubility	Molecular Formula	C_15_H_26_O	C_22_H_24_C_l_N_5_O_2_	C_17_H_21_NO_4_	C_18_H_19_N_3_O	C_17_H_21_NO	C_23_H_21_F_7_N_4_O_3_
	Molecular Weight (g/mol)	222.37	425.91	303.35	293.36	255.35	534.43
	HBA	1	3	5	2	2	12
	HBD	1	2	1	0	0	2
	MR	74.00	124.08	83.48	87.39	79.10	118.82
Pharmacokinetics	GI absorption	High	High	High	High	High	Low
	BBB permeate	Yes	Yes	No	Yes	Yes	No
	CYP_1_A_2_ inhibitor	Yes	No	No	Yes	Yes	No
Drug‐likeness	Ghose	Yes	Yes	Yes	Yes	Yes	No
	Lipinski	Yes	Yes	Yes	Yes	Yes	Yes
	Veber	Yes	Yes	Yes	Yes	Yes	Yes
	BS	0.55	0.55	0.55	0.55	0.55	0.55

NDL: Nerolidol, D_1_‐D_2_: Dopaminergic, 5HT_3_: Serotoninergic, H_1_: Histaminergic, NK_1_: Neurokinin type 1, M_1_‐M_5_: Muscarinic, GIT: Gastrointestinal tract, BBB: Blood‐brain barrier, BS: Bioavailability score, MW: Molecular weight (g/mol)(optimum =≤500), MF: Molecular formula, HBA: Hydrogen bond acceptor (optimum =≤10), HBD: Hydrogen bond donor (optimum =≤5), and MR: Molar refractivity (optimum =≤140).

#### 2.2.5 *In Silico* Toxicity of the Selected Compounds

**Figure 6:**
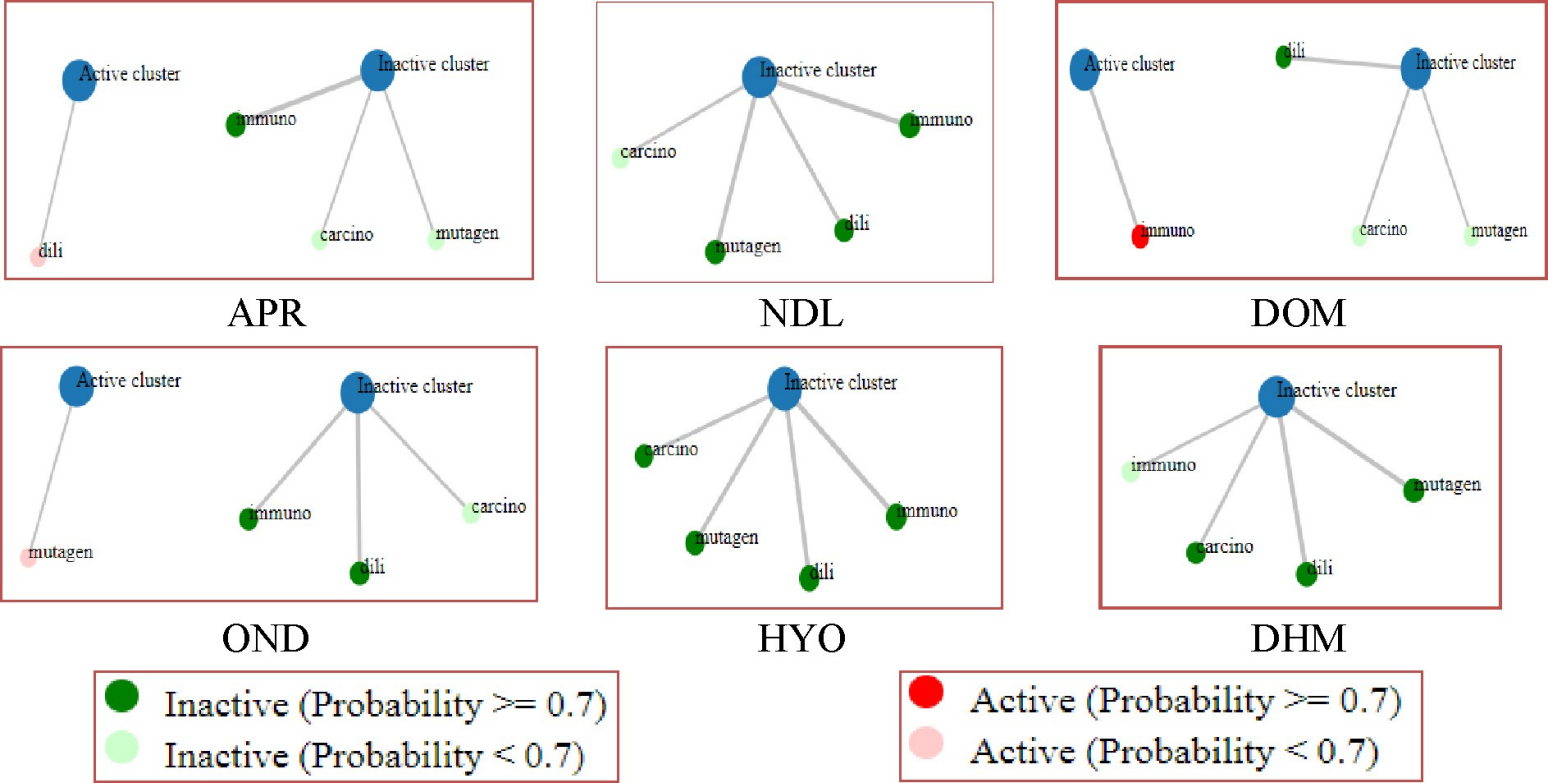
The network chart is intended to rapidly demonstrate the connection among the selected compounds (NDL, APR, HYO, OND, DOM, and DHM) and predicted activities. [mutagen: Mutagenicity; carcino: Carcinogenicity; immuno: Immunotoxicity; dili: Hepatotoxicity; NDL: Nerolidol; APR: Aprepitant, DOM: Domperidone, DHM: Diphenhydramine, HYO: Hyoscine, OND: Ondansetron.]

ProTox 3.0, an online website, is used to measure toxicity parameters. In our *in silico* toxicity results, NDL exposes a lethal dose 50 (LD_50_) of 5000 mg/kg body weight with toxicity classes that are given in Table 5 and Figure 6. Besides, NDL exhibited no toxicity effects in cases of hepatotoxicity, carcinogenicity, immunotoxicity, or mutagenicity. On the other hand, standard drugs, DOM and OND, show immunotoxicity and mutagenicity and APR shows hepatotoxicity.

**Table 5:** The toxicity prediction of nerolidol and other standards using the ProTox 3.0 model.

Properties	Parameters	NDL	DOM	HYO	OND	DHM	APR
Toxicity	LD_50_	5000 mg/kg	715 mg/kg	1275 mg/kg	95 mg/kg	64 mg/kg	1700 mg/kg
	Toxicity class	5	4	4	3	3	4
	Hepatotoxicity	Inactive	Inactive	Inactive	Inactive	Inactive	Active
	Carcinogenicity	Inactive	Inactive	Inactive	Inactive	Inactive	Inactive
	Immunotoxicity	Inactive	Active	Inactive	Inactive	Inactive	Inactive
	Mutagenicity	Inactive	Inactive	Inactive	Active	Inactive	Inactive

LD_50_: Lethal dose‐50; NDL: Nerolidol; APR: Aprepitant, DOM: Domperidone, DHM: Diphenhydramine, HYO: Hyoscine, OND: Ondansetron.

## 3 Discussion

Insufficient control of vomiting can directly deprive treatment observation. The causes of vomiting are a combination of the method of administration, the chemotherapeutic system, experiences, etc..^[38]^ Noxious CuSO_4_, which is consumed orally, can stimulate a specific vegal‐induced vomiting response that can damage the GIT's mucous membrane. It's happened because of its effectiveness as a corrosive and oxidizing agent in CuSO_4_.[^39^, ^40^] Because of the outcome of peripheral mechanisms, it causes the initiation of emesis. By these peripheral methods, visceral afferent nerve fibers of the GIT are triggered and cause the transmission of these stimuli towards the VC.[^41^, ^42^, ^43^] The CTZ in the medulla is the prime mediator of emesis. It operates by initiating a second VC area. However, some receptors such as D_2_‐D_3_,^[44]^ 5HT_3_,^[45]^ H_1_,^[46]^ M_1_‐M_5_ receptors,^[47]^ OP receptors,^[48]^ and cannabinoid (CB) receptors,^[49]^ etc. are used to stimulate emesis in CTZ.

At the CTZ placed in the brain, D_2_ and D_3_ receptors resulted in a lessening of symptoms by counteracting the effects. Its mechanism of action includes the antagonism or inhibition of these receptors. Our selected standard medication, DOM, showed peripheral selective antagonists for these receptors. It is a prokinetic drug that impacts the upper GIT. This reference drug is broadly used for nausea and vomiting treatment.[^6^, ^50^, ^51^] Additionally, our chosen APR is an extremely selective antagonist of the NK_1_ receptor. It is also used for the treatment and management of postoperative (PP) and chemotherapy‐induced (CTI) vomiting and nausea. It causes central blockage in the AP, visceral afferent nerves, and nucleus tractus solitaries.^[52]^ However, the 5HT_3_ receptor also has antiemetic activity by targeting GIT and CTZ in the central nervous system (CNS). 5HT_3_ antagonists such as OND prevent CTI and PP nausea and vomiting and cause relief from vomiting.^[53]^ Furthermore, H_1_ plays a significant role in transferring signals from the GIT, which leads to queasiness. DHM is an antihistamine drug that plays an essential role by antagonizing the H_1_ receptor and reducing emesis.^[54]^

In our *in vivo* experiment, we observed the latency and number of retching for each group of chicks who received the DOM, APR, DHM, OND, HYO, and our tested compound, NDL. The DOM group exposed a mean value of 11.17±1.78 number of retches, whereas the number of retches in the control group was 71.33±4.96, which was a higher decrease in retching than DOM. In addition, DOM outstandingly increased the latency period (74.50±5.66 sec) in comparison to the control group (8.33±0.99 sec), which proves that this drug has antiemetic activity. The administration of DHM, HYO, and OND reduced the retches and increased the latency in the chick group compared to the control group. According to the outcomes, it is promising to assume that NDL established a defensive power against poison by preventing or justifying neural signals that are liable for showing emetic reactions. Significant mitigation in the number of retches in groups NDL‐50 and NDL‐100 was observed, and the obtained mean values were 36.67±3.35 and 29.17±3.25, respectively, in comparison to the control group. In contrast, the APR, DOM, DHM, HYO, and OND groups administered to the animals exhibited higher latency times compared to the control group. Particularly, the latency periods were 9.67±1.84, 74.50±5.66, 11.00±2.05, 16.67±2.81, and 21.83±1.85 sec for standard drugs, APR, DOM, DHM, HYO, and OND, respectively. Surprisingly, the NDL‐100 group of treatments exposed the highest latency period amongst other standard drugs. That's why it exhibited that the treatment group of NDL‐100 is more effective and powerful in augmenting the latency period and declining the retches in CuSO_4_.5H_2_O‐induced emesis in comparison to the other standard groups of treatment.

**Figure 7:**
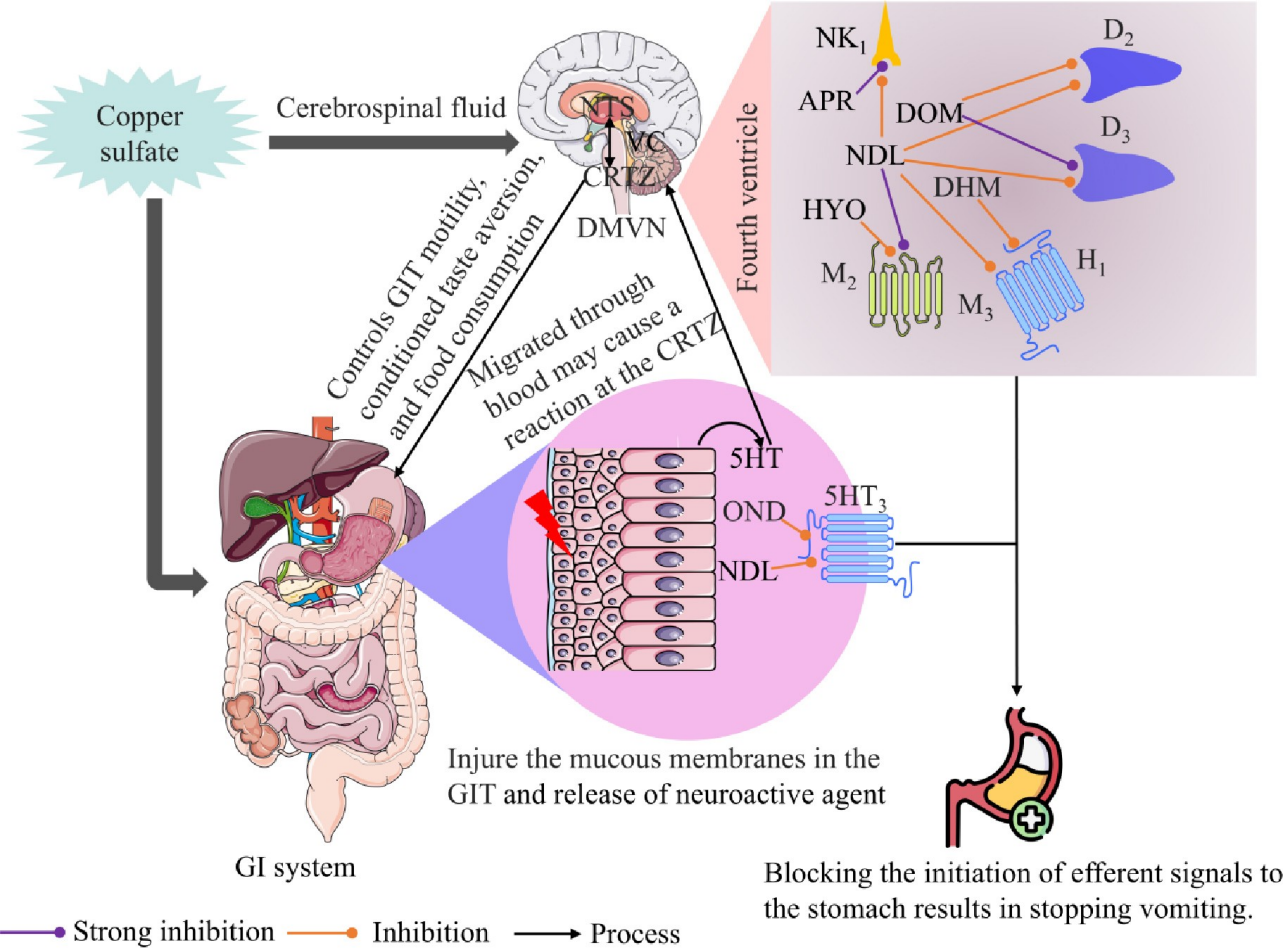
The predicted antiemetic mechanism of the test sample (nerolidol) for the selected standard medications. D_2_ is the dopamine receptor; M_2_ and M_3_ are the muscarinic receptor; CTZ is the chemoreceptor trigger zone; 5HT is the 5‐hydroxy tryptamine; and 5HT_3_ is the serotonin receptor 3. According to their affinity binding to the D_2_, D_3_, 5HT_3_, M_3_, and M_2_ receptors, DOM, OND, and HYO have anti‐emetic mechanisms, while NDL may also have one. These processes are depicted in this figure. In this case, DOM, OND, and HYO block D_2_, D_3_, 5HT_3_, M_2_, and M_3_ receptors, while NDL inhibits D_2_, D_3_, 5HT_3_, M_2_, and M_3_ receptors. [When these stomach receptors are inhibited, the GIT does not contract, and there is no emesis because the VC is restrained from being triggered.]

Synergism, also known as synergistic effects, is the outcome of various drugs working together more efficiently than they would separately when taken independently in the discipline of pharmacology.^[55]^ So, the combination drug therapy demonstrated remarkable outcomes and a synergistic effect, which resulted in a longer latency time and lower retches in experimental animals.^[56]^ In our *in vivo* study, the combination therapies of DOM+NDL‐100, HYO+NDL‐100, and OND+NDL‐100 exhibited a significant increase in latency and a decrease in retches compared to the control group. Specifically, DOM+NDL‐100 group therapy showed an incredible result among all treatment groups. Additionally, the percentage increase in latency and decrease in retches of the combined therapy (DOM+NDL‐100) were 89.45 and 79.67 %, respectively. On the other hand, the result found that, as an outcome of vagal nerve stimulation, CuSO_4_‐mediated emesis does not happen. It was noticed that emesis could not be prohibited even after performing a vagotomy that included cutting the last part of the vagus nerve in the GIT.^[14]^ Depicted in Figure 7 is the recommended anti‐emetic mechanism of the standard medications APR, DOM, DHM, HYO, and OND, and the tested compound, NDL.

A computational methodology, molecular docking, has become an essential element of the drug discovery method. It is applied to inspect the ligand and receptor binding ability, develop a better technique, and carry out it for both energetic and geometric criteria into contemplation.[^57^, ^58^, ^59^] This inspection has made it achievable to screen, create, and improve drug candidates on a new path. To measure the level of interaction between a target ligand and protein, molecular affinity is conducted.[^60^, ^61^] From our *in silico* study, the binding interactions between the NDL and M_2_ receptors were higher in comparison to other receptors that are engaged in the initiation of emesis. The interaction between NDL and M_3_ also manifested the same binding affinity result. The binding energies of NDL through M_2_ and M_3_ were −7.3 kcal/mol for both. The interaction between ligand and protein visualization demonstrates that the binding regions were SER107, CYS429, VAL111, ALA194, TYR104, TRP155, TRP400, TYR403, TYR426, and TYR430 for M2 and NDL interaction; and SER151, CYS532, VAL155, ALA238, TYR148, TRP199, TRP503, TYR506, TYR529, and TYR533 for M_3_ and NDL interaction (including all types of bonds). Additionally, the binding energy of NDL with D_2_ and D_3_ receptors was −6.5 kcal/mol for both, which is also greater than H_1_, M_1_, M_4_, M_5_, and 5HT_3_ receptors. On the other hand, in comparison to the combined AA residues of several receptors amid the ligand NDL and the chosen drugs, after that, the AA residues of VAL107 for D_3_, LEU129 and HIS134 for 5HT_3_, TYR426 for M_2_, TYR148, CYS532, TYR148, TRP503, TYR506, TYR529, and ALA238 for M_3_, TRP477 for M_5_, HIS265, HIS197, TRP261, and PHE264 for NK_1_, PHE116, PHE119, PRO202, ILE120, and ALA151 for H_1_ are indistinguishable. It explains that, by interaction with the identical AA residue, they are combined at the same site on the receptors. However, the reason for the greater binding affinity of NDL aligned with M_2_, M_3_, and D_3_ receptors is because of the HB arrangement and variety of HPB. In contrast, there is no HB located in the interaction of NDL with M_1_, M_4_, M_5_, and H_1_ receptors. For all of this reason, it can be presumed that NDL exposes greater inhibitory potency for the M_2_, M_3_, D_2_, and D_3_ receptors in comparison to the other receptors liable for emesis.

When searching for novel drugs, scientists find a common dilemma: low oral bioavailability.^[62]^ Our chosen standard compound (NDL) also has the same problem. According to some review papers, NDL showed a lower peak plasma concentration in cases of oral administration than intraperitoneal administration in animals.^[63]^ It happens because oral administration of NDL must first undergo first‐pass action, meaning that it must pass via the intestinal wall and then to the liver and portal circulation.^[64]^ Thus, a bit of the oral dose of NDL vanished in the first‐pass metabolism, contributing to lower bioavailability in comparison to the intraperitoneal route of administration.^[65]^ Additionally, NDL is a lipophilic particle that can penetrate into the BBB. However, improvement of passage via the BBB can be done by nanotechnology using NDL‐loaded nanospheres.^[66]^ Furthermore, the study has exposed that the instantaneous co‐treatment of NDL over the BBB leads to an increase in the movement of NDL.^[67]^

Specific laboratory animals are used for *in vivo* studies that give vital information on the optimistic and unenthusiastic actions of new drugs with probable biopharmaceutical issues. Therefore, in measuring the possibility of biologically active compounds for clinical trials, every pre‐clinical analysis supports medical researchers.^[68]^ Our study exhibited limitations based on the behavioral depiction of the animals, such as the deficiency of clinical trials and outcomes. This study is based on *in vivo* and *in silico* studies that show a possible antiemetic mechanism of NDL. Putting all of the information together, our findings revealed that NDL exhibits a potent antiemetic effect in experimental animals by augmenting the latency and lowering the number of retching of emesis. Additionally, the *in silico* investigation demonstrated the causes following the antiemetic actions of NDL, possibly via the connection of NDL with dopaminergic, serotonergic, and muscarinic receptors.

## 4 Conclusions

In recapitulation, the outcomes of our study confirm that NDL shows prominent antiemetic activity and mitigates CuSO_4_.5H_2_O‐mediated retching in chicks efficiently through its peripheral action. Additionally, *in silico* studies have also shown possible antiemetic effects with greater binding affinity against muscarinic (specially, M_2_ and M_3_) and dopaminergic receptors. In total, NDL also exhibits synergistic effects when given in an amalgamation with well‐known antiemetic drugs through binding affinity. As a result, NDL might be an optimistic one in the treatment of emesis. However, more research with animals and rational studies (using human organs on chips) are required to evaluate the precision and efficacy of NDL. In addition, it is not clear how NDL slows down the emesis precisely, while the molecular routes for emesis may have been fully understood. That's why additional research is needed to establish an accurate and efficient mechanism of action for NDL in treating nausea and vomiting.

## 5 Materials and Methods

### 5.1 *In Vivo* Study

#### 5.1.1 Chemicals and Reagents

NDL (*3,7,11‐trimethyl‐1,6,10‐dodecatrien‐3‐ol*), 98 %, and a mixture of *cis* and *trans* isomers (CAS Number: 7212–44‐4) were purchased from Sigma‐Aldrich (USA), while copper sulfate pentahydrate (CuSO_4_.5H_2_O) and tween 80 were purchased from Merck (India). The mentioned drugs, domperidone (DOM), ondansetron (OND), hyoscine butylbromide (HYO), aprepitant (APR), and diphenhyramine (DHM), were collected from Beximco, Incepta, Opsonin, Beacon and Eskayef Pharma Ltd., Bangladesh, respectively.

#### 5.1.2 Selection and Preparation of Doses

According to some research papers, we selected two concentrations of the test sample: one lower and another higher. We arranged the mother solution of the sample at a concentration of 100 mg/kg by dissolving it in distilled water (DW). A small amount of Tween 80 (0.5 %) is used as a co‐solvent. To get the 50 mg/kg concentration, the mother solution was diluted. On the contrary, the reference drug doses were taken by changing human doses to animal doses, supported by the protocol of animal dose calculation and literature methods.[^14^, ^69^] The solutions of the mentioned drugs were also prepared by mixing carefully into DW (also using a small amount of Tween 80) at 16, 6, 10, 21, and 5 mg/kg concentrations for the APR, DOM, DHM, HYO, and OND drugs **(**Table 6 **)**. Additionally, three mixed doses were prepared for the co‐treatment: NDL (100 mg/kg), with DOM, HYO, and OND. It helped to know about the synergistic or antagonistic effects of the combination therapies.

**Table 6:** Different treatments and their doses were investigated in animals.

Treatment Groups	Composition	Dose(mg/kg)	Target Receptor
Control	Vehicle (0.5 % Tween 80 dissolved in DW)	10 ml/kg	‐
APR	Aprepitant	16	NK_1_
DOM	Domperidone	6	D_2_
DHM	Diphenhydramine	10	H_1_
HYO	Hyoscine Butylbromide	21	M_1_‐M_5_ muscarinic acetylcholine
OND	Ondansetron	5	5HT_3_
NDL‐50	Nerolidol	50	Under Investigation
NDL‐100		100	
DOM+NDL‐100	Domperidone+Nerolidol	6+100	Under Investigation
HYO+NDL‐100	Hyoscine Butylbromide+Nerolidol	21+100	Under Investigation
OND+NDL‐100	Ondansetron+Nerolidol	5+100	Under Investigation

#### 5.1.3 Experimental Animals

We purchased young chicks (*Gallus gallus domesticus*) of both genders with a 40–46 g weight range, 2 days old, from Provita Feed and Hatcheries Ltd. at Road‐3, House‐270, Baridhara DHOS, Dhaka, 1206, Bangladesh. Chicks were reserved at Bangabandhu Sheikh Mujibur Rahman Science and Technology University's pharmacology lab, Gopalganj, for this experiment. They were conserved at 27±2 °C with a 12‐hour dark/light cycle under controlled illumination before the experiment began. After 12 hours of fasting, the antiemetic test was conducted. This experiment was approved by the Department of Pharmacy and the Ethical Committee of Bangabandhu Sheikh Mujibur Rahman Science and Technology University. Besides, all techniques were carried out following applicable guidelines and regulations (#bsmrstu‐pharmacy‐18PHR019). All procedures were reported in agreement with ARRIVE guidelines (https://arriveguidelines.org).

#### 5.1.4 *In Vivo* Protocols

The experiment was carried out with slight adjustments to the techniques narrated by Akita et al. (1998).^[70]^ All the chicks were divided into eleven groups, with six in each. Before starting the experiments, each chick was placed in a large, transparent plastic carrier for 10 minutes. Two doses (50 and 100 mg/kg) of the test sample (NDL) were prepared with DW and orally given with the help of a literature review. DOM, OND, and HYO were administered as reference drugs at doses of 6, 5, and 21 mg/kg b.w. orally and correspondingly. Three combined doses of the reference drugs were prepared by mixing them with NDL (100 mg/kg) and administered to animals orally to calculate and estimate their synergistic effect. DW was given orally at a dose of 10 mg/kg b.w., which was considered a negative control. After 30 minutes of treatment, the CuSO_4_.5H_2_O inducer was given orally at a dose of 50 mg/kg. After that, the number of retches (within 10 minutes after taking CuSO_4_.5H_2_O treatment) and latency (first retches after having CuSO_4_.5H_2_O) were recorded precisely. Then the percentage decrease in retches and increase in latency compared to the control group were calculated based on the following equations:

$$\%\;\mathrm{increase}\;\mathrm{in}\;\mathrm{latency}=\;\frac{X-Y}{X}\times 100\;\%$$

$$\%\;\mathrm{decrease}\;\mathrm{in}\;\mathrm{retches}=\frac{R-P}{R}\times 100\;\%$$

Here, X=mean of latency in seconds in standard and test groups, Y=mean of latency in seconds in the control group, R=mean of retches in the control group, and P=mean of retches in standard and test groups.

#### 5.1.5 Statistical Analysis

The antiemetic effects values are reported as the mean value, along with the standard error of the mean (SEM). Graph Pad Prism (Version 6), a statistical software program, was used to estimate the variance's statistical significance, which was determined using a 95 % confidence interval. *p* values of <0.05 were considered significant.

### 5.2 *In Silico* Analysis

#### 5.2.1 Homology Model and Preparation of Receptors

According to some research papers, we selected ten receptors to construct docking molecules and ligand‐receptor visualization. We established a homology model due to the unavailability of the 3D structure of human 5HT_3_ receptors in the RCSB Protein Data Bank.^[71]^ Human 5HT_3_ homology modeling was developed via the use of SWISS‐MODEL.^[72]^ The UniProt database was used to regain the sequence of proteins and to find the best template, the NCBI BLAST program was used to establish a BLAST analysis.^[73]^ By using MolProbity^[74]^ for the Ramachandran plot, we verified and evaluated the 5HT_3_ homology modeling structures. D_2_ (PDB ID: 6LUQ),^[75]^ D_3_ (PDB ID: 3PBL),^[76]^ H_1_ (PDB ID: 3RZE),^[77]^ M_1_ (PDB ID: 6WJC), M_2_ (PDB ID: 5ZK8), M_3_ (PDB ID: 4 U15), M_4_ (PDB ID: 7 V6 A), M_5_ (PDB ID: 6OL9),^[78]^ and NK_1_ (PDB ID: 6HLO)^[79]^ were acquired from the RCSB Protein Data Bank (website link: https://www.rcsb.org/). After that, we used the PyMol software program (v3.0.3) to eliminate any irrelevant molecules, such as water, lipids, heteroatoms, etc., from the protein sequence to optimize the receptors and avert docking obstructions. In the end, utilizing the SWISSPDB viewer software program, the shape and energy of receptors were minimized. Then the PDB file was saved for the next step, molecular docking.

#### 5.2.2 Collection and Preparation of Ligands

**Figure 8:**
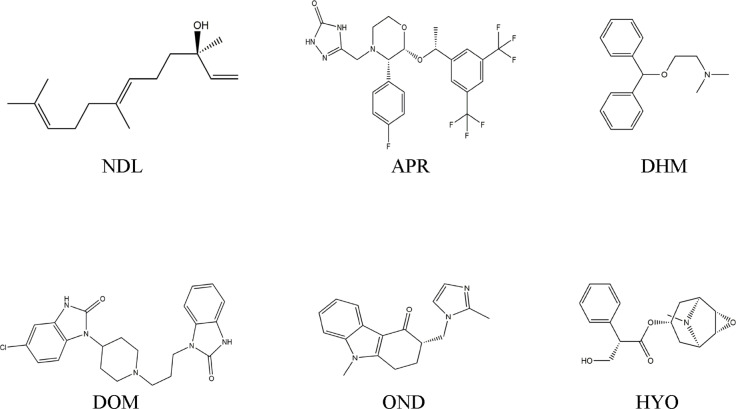
2D Structures of nerolidol and selected reference drugs screened against the emesis‐inducing receptors.

According to the literature, we selected well‐known and marketable antiemetic medicines as reference ligands in comparison to the binding energy and molecular interaction of our test ligand (NDL). It was highlighted in several emesis‐causing receptors to know the main cause of the anti‐emetic mechanism. After that, various receptors and the determination of PK types of the 3D conformers of NDL (Compound CID: 5284507), APR (Compound CID: 135413536), DOM (Compound CID: 3151), HYO (Compound CID: 3000322), DHM (Compound CID: 3100), and OND (Compound CID: 4595) were collected from the PubChem chemical database in SDF format (https://www.ncbi.nlm.nih.gov/). Next, we used the Chem3D 16.0 computer application that is utilized for executing molecular docking and predicting PK. Through this application, the 3D conformers of the chemical substance were minimized and saved in SDF format. The optimization was done in the PyRx app before docking began. The chemical structures of NDL and standard drugs are given in Figure 8.

#### 5.2.3 Molecular Docking study

Using the PyRx application, molecular docking was organized to predict the active binding energy of the drugs with the active sites of receptors. For exploring binding sites and poses of receptors and ligands, blind docking was used with a maximum grid box dimension according to protein‐ligand complex size^[80]^ and 500 steps of steepest descent.^[81]^ The docking potential outcome is saved in ‘csv’ format, and the ligand‐protein complex file is gleaned in PDB format to collect the PDBQT format's ligand. The interaction between ligand‐receptors was seen via the PyMol computer application (version 3.0.3) and Discovery Studio Visualizer (version 24.1.0). After that, the number and length of HB, HPB, AA residues, etc. are predicted.

#### 5.2.4 Prediction of Drug‐Likeness and Pharmacokinetics

A qualitative measurement that is drug‐likeness is used to examine the potentiality of molecules to be improved and discovered into a medication taken orally. To demonstrate similarities between the substances and existing drugs, a physicochemical analysis was conducted.^[82]^ In this study, we used the SwissADME website to understand, evaluate, examine, and predict the physicochemical characteristics of the test substance (http://www.swissadme.ch/).

#### 5.2.5 Toxicity Prediction

For the estimation of several toxicity parameters of any substance, ProTox 3.0 online servers can be used. It is utilized to measure the safety profile of a compound by inspecting hepatotoxicity, mutagenicity, carcinogenicity, and other toxicity parameters.^[83]^ For this, we entered Canonical SMILES into this online server that was gleaned from PubChem (https://tox.charite.de/protox3/). The toxicity parameters of the chosen compounds are given in Table 5.

## Author Contributions

All authors made a significant contribution (conceptualization, methodology, software, formal analysis, writing–original draft preparation, writing–review and editing, validation, and supervision) to the work. All authors have read and agreed to the published version of the manuscript.

6

## Conflict of Interests

The authors declare no conflict of interest.

## Data Availability

Not applicable.
